# Editorial: MR Spectroscopy in Neuropsychiatry

**DOI:** 10.3389/fpsyt.2018.00197

**Published:** 2018-05-31

**Authors:** Alice Egerton, Anouk Marsman, Brian V. Broberg, Hilleke E. Hulshoff Pol

**Affiliations:** ^1^Department of Psychosis Studies, King's College London, Institute of Psychiatry, Psychology and Neuroscience, London, United Kingdom; ^2^Danish Research Centre for Magnetic Resonance, Centre for Functional and Diagnostic Imaging and Research, Copenhagen University Hospital Hvidovre, Hvidovre, Denmark; ^3^Centre for Neuropsychiatric Schizophrenia Research, Centre for Clinical Intervention and Neuropsychiatric Schizophrenia Research, Mental Health Centre Glostrup, Mental Health Services, Capital Region of Denmark, University of Copenhagen, Glostrup, Denmark; ^4^Department of Psychiatry, Brain Center Rudolf Magnus, University Medical Center Utrecht, Utrecht, Netherlands

**Keywords:** MR spectroscopy, neurochemistry, neuroimaging, schizophrenia, glutamate, GABA, 7 tesla MRI, functional MRS

Magnetic resonance spectroscopy (MRS) is a non-invasive neuroimaging technique with the unique ability to reveal the role of neurometabolites in brain physiological and pathophysiological processes. MRS-visible metabolites include the neurotransmitters glutamate and GABA, as well as compounds implicated in oxidative stress or inflammation, such as glutathione and myo-inositol. Optimal application of MRS techniques therefore has significant potential for revealing brain pathophysiological mechanisms in psychiatric disorders.

The last decade has seen substantial developments in MRS capability. These advances have been driven by the availability of high field strength MR scanners, in parallel with new MRS acquisition and data analysis methods. It is now possible to accurately detect previously indistinguishable MRS metabolites, such as GABA and glutathione, or to measure dynamic changes in metabolite levels occurring during behavioral challenges or in response to other stimuli. The articles in this research topic together provide an update on the current state-of-the-art MRS methodology, and, using schizophrenia as an example, illustrate how MRS may be applied to increase understanding of neurochemical abnormalities across the psychiatric disorders.

Three articles in this research topic focus on current state-of-the-art MRS methodology. Godlewska et al. provide an overview of ultra-high-field (UHF) MRS. In UHF MRS, data are acquired at MR field strengths of 7 Tesla (7T) or more, resulting in high sensitivity and resolution, and allowing quantification of numerous metabolites in the MR spectra. This review provides a practical guide to the advantages and disadvantages of UHF MRS, together with examples of its application in psychiatric disorders. Development of UHF methodology is further described in the article of Marsman et al. which presents a comparison of two different acquisition sequences for glutamate quantification at 7T; the more common approach of STEAM vs the recent approach of sLASER. Comparing test-retest reproducibility in healthy volunteers, this study demonstrates advantage of sLASER acquisition in robustly and sensitively determining glutamate levels in the frontal cortex.

In the third article of the research topic focussing on methodology, Stanley and Raz describe the emerging and innovative approach of functional MRS (fMRS). Typically, MRS is used to measure metabolite levels while the subject is at rest in the scanner. In contrast, fMRS can measure the dynamic changes in metabolites, particularly glutamate, which occur while subjects perform a behavioral task. This review provides a summary of the technological advances, strengths and limitations of fMRS, and describes how this approach is contributing to our understanding of the glutamatergic basis of cognitive impairments in psychiatric disorders.

Marsman et al. then bring together the themes of UHF MRS and the role of glutamate in cognition in their study at 7T examining the relationship between working memory and the level of glutamate and GABA in the frontal and occipital cortex. The results indicate that working memory performance in healthy individuals is related to the balance between these excitatory and inhibitory neurotransmitters.

The remaining articles in the research topic relate to schizophrenia, and provide complementary examples of MRS application in psychiatric research. A principal focus of MRS research in schizophrenia has been on the measurement of brain glutamate levels, due to its core implication in schizophrenia pathophysiology. Bustillo et al. provide a significant step forward, by combining glutamate genetic data with MRS imaging in schizophrenia, and thereby linking two fields of glutamatergic research. This study found that in younger patients with schizophrenia, the combined score for three single nucleotide polymorphisms in glutamatergic genes associated with schizophrenia risk partially contributes to elevated levels of Glx (the combined MRS signal from glutamate and glutamine) in gray matter. Interestingly this association was not apparent in older patients, which suggests that other factors affect Glx levels during the course of schizophrenic illness.

There is some debate as to whether antipsychotic medication may alter brain glutamate levels, and the article of Egerton et al. provided a systematic review of longitudinal MRS studies that have addressed this question. Although these studies have used a range of methodological approaches and have investigated different brain regions, the majority report a numerical reduction in Glx levels over the course of antipsychotic treatment, suggesting that to at least some degree antipsychotics may reduce glutamate elevation in schizophrenia.

The study of Bojesen et al. addresses potential associations between schizophrenia-related abnormalities in glutamate levels and brain activity, measured as regional cerebral blood flow (rCBF). To avoid the potential confounds of medication or prolonged illness that may be present in a patient sample, this study used administration of the N-methyl-D-aspartate (NMDA) antagonist ketamine in healthy volunteers as a glutamatergic experimental model of schizophrenia pathophysiology. The observed associations between glutamate and rCBF in the anterior cingulate cortex may suggest a mechanistic link between these neurobiological features relevant to schizophrenia.

The development of MRS techniques to accurately measure GABA has permitted investigation of GABA abnormalities in schizophrenia *in vivo*, which were previously mainly understood on the basis of post-mortem data. The review of de Jonge et al. summarizes the findings to date from GABA MRS studies in relation to post-mortem evidence, and suggests key future directions for this emerging field. Finally, included in this research topic is the study of Rowland et al. which used levels of the MRS metabolites myo-inositol and choline-containing compounds as an innovative proxy measure of brain inflammation in schizophrenia. This exploratory study found correlations between antiglandin antibody levels and the MRS “inflammation measures,” and may open new avenues for MRS application in testing inflammatory theories of brain disorders.

Through these examples from schizophrenia research, these articles demonstrate how MRS can be applied to increase our understanding of neurochemical abnormalities or inflammation across neurological and psychiatric disorders. Recent advances in MRS technology will play a major role in achieving this goal, and this research topic provides a practical guide to the emerging MRS technologies of UHF MRS and fMRS. Together these articles provide a resource for researchers interested in MRS approaches and understanding their strengths and limitations, and also some inspiration for future research applications.

## Author contributions

All authors listed have made a substantial, direct and intellectual contribution to the work, and approved it for publication.

### Conflict of interest statement

The authors declare that the research was conducted in the absence of any commercial or financial relationships that could be construed as a potential conflict of interest.

